# Neuroprotective potential of *Marsilea quadrifolia* Linn against monosodium glutamate-induced excitotoxicity in rats

**DOI:** 10.3389/fphar.2023.1212376

**Published:** 2023-09-14

**Authors:** Arunkumar Subramanian, T. Tamilanban, Mahendran Sekar, M. Yasmin Begum, Akhtar Atiya, Gobinath Ramachawolran, Ling Shing Wong, Vetriselvan Subramaniyan, Siew Hua Gan, Nur Najihah Izzati Mat Rani, Yuan Seng Wu, Suresh V. Chinni, Shivkanya Fuloria, Neeraj Kumar Fuloria

**Affiliations:** ^1^ Department of Pharmacology, SRM College of Pharmacy, SRM Institute of Science and Technology, Chengalpattu, Tamil Nadu, India; ^2^ School of Pharmacy, Monash University Malaysia, Subang Jaya, Selangor, Malaysia; ^3^ Department of Pharmaceutics, College of Pharmacy, King Khalid University, Abha, Saudi Arabia; ^4^ Department of Pharmacognosy, College of Pharmacy, King Khalid University (KKU), Abha, Saudi Arabia; ^5^ Department of Foundation, RCSI & UCD Malaysia Campus, Georgetown, Pulau Pinang, Malaysia; ^6^ Faculty of Health and Life Sciences, INTI International University, Nilai, Malaysia; ^7^ Jeffrey Cheah School of Medicine and Health Sciences, Monash University Malaysia, Subang Jaya, Selangor, Malaysia; ^8^ Center for Transdisciplinary Research, Department of Pharmacology, Saveetha Dental College, Saveetha Institute of Medical and Technical Science, Saveetha University, Chennai, India; ^9^ Faculty of Pharmacy and Health Sciences, Royal College of Medicine Perak, Universiti Kuala Lumpur, Ipoh, Perak, Malaysia; ^10^ School of Medical and Life Sciences, Sunway University, Subang Jaya, Malaysia; ^11^ Department of Biochemistry, Faculty of Medicine, Bioscience, and Nursing, MAHSA University, Selangor, Malaysia; ^12^ Department of Periodontics, Saveetha Dental College and Hospitals, Saveetha Institute of Medical and Technical Sciences, Saveetha University, Chennai, India; ^13^ Faculty of Pharmacy, AIMST University, Bedong, Kedah, Malaysia

**Keywords:** *Marsilea quadrifolia* Linn, excitotoxicity, neuroprotection, quercetin, antioxidant

## Abstract

**Background:** Excitotoxicity is a condition in which neurons are damaged/injured by the over-activation of glutamate receptors. Excitotoxins play a crucial part in the progression of several neurological diseases. *Marsilea quadrifolia* Linn (*M. quadrifolia*) is a very popular aquatic medicinal plant that has been utilised for a variety of therapeutic benefits since ancient times. Its chemical composition is diverse and includes phenolic compounds, tannins, saponins, flavonoids, steroids, terpenoids, alkaloids, carbohydrates and several others that possess antioxidant properties.

**Objective:** The objective of the present study was to investigate the neuroprotective potential of *M. quadrifolia* against monosodium glutamate (MSG)-induced excitotoxicity in rats.

**Methods:** A high-performance thin-layer chromatography (HPTLC) analysis of chloroform extract of *M. quadrifolia* (CEMQ) was conducted to identify the major constituents. Further, the *in silico* docking analysis was carried out on selected ligands. To confirm CEMQ’s neuroprotective effects, the locomotor activity, non-spatial memory, and learning were assessed.

**Results and discussion:** The present study confirmed that CMEQ contains quercetin and its derivatives in large. The *in-silico* findings indicated that quercetin has a better binding affinity (−7.9 kcal/mol) towards the protein target 5EWJ. Animals treated with MSG had 1) a greater reduction in the locomotor score and impairment in memory and learning 2) a greater increase in the blood levels of calcium and sodium and 3) neuronal disorganization, along with cerebral edema and neuronal degeneration in the brain tissues as compared to normal control animals. The changes were however, significantly improved in animals which received standard drug memantine (20 mg/kg) and CEMQ (200 and 400 mg/kg) as compared to the negative control. It is plausible that the changes seen with CEMQ may be attributed to the N-methyl-D-aspartate (NMDA) antagonistic properties.

**Conclusion:** Overall, this study indicated that *M. quadrifolia* ameliorated MSG-induced neurotoxicity. Future investigations are required to explore the neuroprotective mechanism of *M. quadrifolia* and its active constituents, which will provide exciting insights in the therapeutic management of neurological disorders.

## Graphical Abstract

**Figure:**
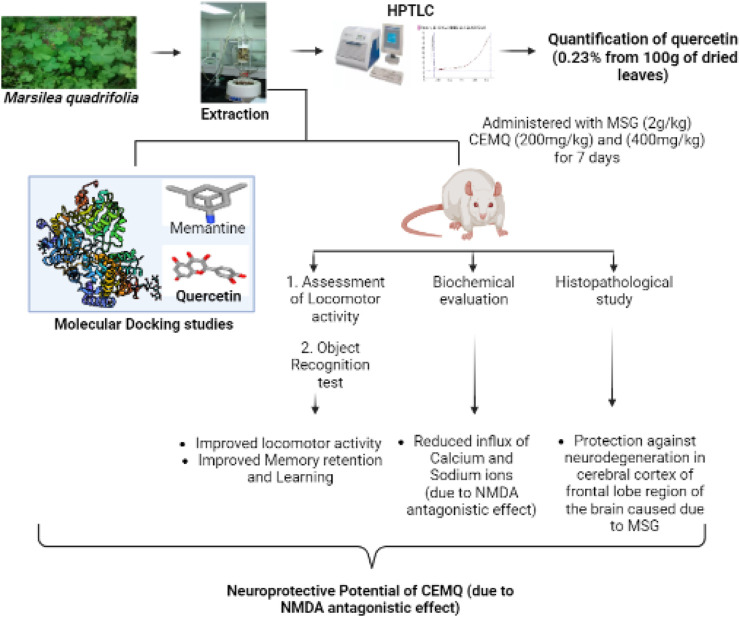

## 1 Introduction

Monosodium glutamate (MSG) is one of the most commonly-used food additives. Although its toxicity has been tested in various animal species, its method of administration and doses are not similar to that for humans. It is said to contribute to obesity as well as what is known as a “Chinese restaurant syndrome” by affecting the central nervous system, adipose tissue, liver and the reproductive systems (Afifi and Abbas, 2011; Singh S. et al., 2021). According to scientific findings, taste enhancers stimulate the taste cells in the tongue and neurons in the brain. Upon exposure, they are exceedingly stimulated and fire impulses quickly until complete exhaustion (Hajihasani et al., 2020) leading to the damage and destruction of neurons occurring via glutamate receptor activation (Rousseaux, 2008). N-methyl-D-aspartate (NMDA), α-amino-3-hydroxy-5-methyl-4-isoxazolepropionic acid (AMPA) and Kainate receptors are the three types of glutamate receptors to which glutamate ions can bind to initiate action. Subsequently, the neurons die due to apoptosis when glutamate concentration in the synaptic cleft can no longer be reduced or reach greater levels. Excitotoxicity is also implicated in the development of a variety of neurological conditions including migraines, seizures, neuropsychiatric disorders, learning disabilities in children and neurodegenerative diseases like Alzheimer’s disease, amyotrophic lateral sclerosis as well as Parkinson’s disease (Heath and Shaw, 2002; Dong et al., 2009; Khalil and Khedr, 2016; Chauhdary et al., 2019; Parambi et al., 2020; Subramanian et al., 2022).

*Marsilea quadrifolia* Linn (*M. quadrifolia*) belongs to the family of Marsileaceae. It grows in the shallow water of the lakes, ponds, and rivers, in many parts of India, China and Southern Europe. The plant is used for its diuretic, anti-inflammatory and depurative properties (Jenila Bejads et al., 2014; Venkatachalam and Balasundaram, 2017). The leaf extract of the plant is also applied externally to treat snake bites and skin injuries (Subramanian and Balakrishnan, 2019). The plant *M. quadrifolia* is traditionally used to treat fever, cold, cough and for wound healing. It contains tannins, flavonoids, betulinic acid, fatty acids and sterols (Gopalakrishnan and Udayakumar, 2017). It is also reported to contain quercetin, hentriacontane and some other important phytoconstituents which have important pharmacological properties (Zhang et al., 2016). Based on the literature (Bhadra et al., 2012) and documentation of the current uses of *M. quadrifolia*, an initiative has been made to verify the scientific validity of investigating its neuroprotective activity. The present study aimed to evaluate the toxic effects of MSG on selected neurobehavioral parameters (non-spatial memory, learning and locomotion), selected region (cerebral cortex of frontal lobe) of brain, fluctuations in selected brain biochemical parameters (sodium and calcium levels) and its reversal by the *M. quadrifolia* leaves extract to claim its neuroprotective potential.

## 2 Materials and methods

### 2.1 Collection and authentication of *M. quadrifolia*

In January 2022, *M. quadrifolia* leaves were collected locally in the wetlands of the Yercaud foothills, Salem, Tamilnadu, India. The plant was taxonomically identified and confirmed by a botanist, Dr. A. Balasubramanian of the ABS Botanical Garden, Salem, Tamilnadu, India.

### 2.2 Extraction

The collected fresh *M. quadrifolia* leaves were washed under running tap water to remove the soil and other dust like contaminants. 100 g of the cleaned and moisture free *M. quadrifolia* leaves were extracted using a continuous hot percolation process with a Soxhlet apparatus for 18–24 h. Various successive solvents based on their increasing polarities were used (starting from petroleum ether, chloroform to ethanol). Subsequently, the extracts were concentrated under controlled temperature and reduced pressure using rotary evaporator. The chloroform extract of *M. quadrifolia* (CEMQ) was then subjected to a series of qualitative chemical tests to determine the type of phytoconstituents present in it (depicted in Table 1).

**TABLE 1:** Qualitative phytochemical screening of CEMQ.

S. No	Constituents	Tests	Present/Absent
1	Carbohydrates	Molisch’s test	Present
		Fehling’s test	
2	Glycosides	Legal’s test	Present
		Borntrager’s test	
		Baljet test	
3	Flavonoids	Lead acetate test	Present
		Con.H_2_SO_4_ test	
		FeCl_3_ test	
4	Alkaloids	Dragendorff’s test	Absent
		Mayer’s test	
		Wagner’s test	
		Hager’s test	
5	Phytosterols	Salkowski test	Present
		Libermann-Burchard test	
6	Phenolic compounds and tannins	FeCl_3_ test	Present
		Lead acetate test	
		Gelatin test	
7	Saponins	Foam test	Present
8	Fixed oil and fats	Spot test	Absent
		Saponification test	

### 2.3 HPTLC analysis

Among the crude extracts, the chloroform leaves extract of the plant was subjected to a high-performance thin-layer chromatography (HPTLC) analysis. The mobile phase composition and detection wavelength were fixed to provide precise, accurate and repeatable findings (Gomathi et al., 2012; Singh S. A. et al., 2021; Shah et al., 2022). The mobile phase consisted of toluene: ethyl acetate: formic acid: water (3:6:0.6:0.4). A pre-coated silica gel (G_60_F_254_ plate) was used as a stationary plate to achieve a good separation of the drugs with good symmetrical peaks. The components’ spot was scanned at 254 nm.

#### 2.3.1 Chamber saturation time

A 5 min saturation period results in good component resolution and peak forms (Bhargava et al., 2021). As a result, the study’s saturation time was set to 5 min accordingly.

#### 2.3.2 Solvents composition in the mobile phase

Taking the resolution of the drug peak shape and retention time (R_f_) value into consideration, toluene: ethyl acetate: formic acid: water (3:6:0.6:0.4) composition was employed as the mobile phase for the separation.

The R_f_ value was calculated using the following formula;

$$R_{f=}\;\frac{Distance\;from\;the\;start\;to\;the\;center\;of\;the\;chromatographic\;spot}{Distance\;of\;the\;solvent\;from\;the\;start}$$

#### 2.3.3 Optimized chromatographic conditions

Stationary phase - Silica gel (G_60_F_254_ plate)
Mobile phase - Toluene: ethyl acetate: formic acid: water (3:6:0.6:0.4)
Chamber saturation time - 5 min
Plate saturation time - 10 min
Distance of solvent front - 80 mm
Number of tracks - 6
Distance between tracks - 16 mm
Slit dimension - 6.00 × 0.45 mm, Micro
Scan speed - 20 mm/s
Scan wavelength - 254 nm

### 2.4 *In-silico* docking study

In the docking study, the binding affinity was calculated in terms of (kcal/mol). Two ligands were considered: Quercetin, present in the selected plant extract (the selected ligand) and memantine (standard ligand). The NMDA glutamate receptor (5EWJ) protein target was downloaded from the Research Collaboratory for Structural Bioinformatics (RCSB) PDB (Protein Data Bank) as a PDB file. NMDA glutamate ionotropic receptor consists of 376 amino acid sequences in four chains A, B, C, D (Stroebel et al., 2016). Then, the charges and hydrogen bonds were added and water molecules were removed from its 3D structure using a BIOVIA Discovery Studio visualizer before being saved in a pdbqt file format. The ligands were downloaded from the PubChem database while the 3D Structure of quercetin and memantine were downloaded as SDF (Structural Data File).

### 2.5 Pharmacological screening

The study was approved by Institutional Animal Ethics Committee, SRM College of Pharmacy (Approval number: SRMCP/IAEC/314/2022). The Institutional Animal Ethics Committee (IAEC) reviewed and sanctioned the permission for the experimental protocols. Healthy Wistar rats, 12–15 weeks old, weighing 180–220 g were used and allowed to acclimatise to the laboratory environment for 1 week. All animals were housed in properly ventilated polypropylene cages at 25°C and 55%–65% relative humidity. The animals were allowed access to commercial pelleted rodents feed and water *ad libitum*.

The rats were categorized into five groups (six animals in each) as mentioned below.

Group I: Normal saline [1% w/v of carboxy methyl cellulose (CMC), i.p.]
Group II: MSG (2 g/kg, i.p.)
Group III: MSG (2 g/kg, i.p.) + Standard Memantine (20 mg/kg, i.p.)
Group IV: MSG (2 g/kg, i.p.) + CEMQ (200 mg/kg, p.o.)
Group V: MSG (2 g/kg, i.p.) + CEMQ (400 mg/kg, p.o.)
Group II-V rats were administered with MSG (2 g/kg, i.p.) for a period of 7 days to induce neurotoxicity. Further, group III-V were treated with standard memantine or with CEMQ, during the experimental duration of 7 days (Creeley et al., 2006; Shivasharan et al., 2013; Viswanatha Swamy et al., 2013; Merve Bayram et al., 2023).

Based on the acute toxicity study (OECD, 2022), LD_50_ value of the chloroform extract of *M. quadrifolia* was found to be 2000 mg/kg body weight (Test No. 425: Acute Oral Toxicity: Up-and-Down Procedure, 2022). The two doses of 200 and 400 mg/kg of CEMQ has been selected to proceed with the further pharmacological study (Sahu et al., 2012).

#### 2.5.1 Effect of CEMQ on locomotor score

The locomotor score was estimated with the help of a digital actophotometer. The apparatus was placed in a properly ventilated and light, sound-attenuated testing room. Each beam interruption generates an electrical impulse that will be indicated on a digital counter. Each animal was investigated for about 5 min (on day 1, 3, 5 and 7) following 2 h of administration of normal saline, MSG, Memantine and the extracts (Shivasharan et al., 2013; Viswanatha Swamy et al., 2013).

#### 2.5.2 Effect of CEMQ on the object recognition test

The test setup consisted of a box made of plywood (80 × 80 cm) with a grid floor that could be easily flushed with hydrogen peroxide after each trial. Briefly, a 40 W lamp was hanged 50 cm above the box to illuminate the apparatus. Two black, 8 cm tall, plywood pieces of varying shapes were used as discriminatory cues (objects to be discriminated). The day before the test (i.e. on the seventh day), the rats were allowed to explore the box (without any object) for 2 min. On the test day (eighth day), in the first trial (T_1_) two similar objects were placed in two opposite corners of the box and the time spent by each rat to explore the objects was observed.

Exploration was considered as directing the nose at a distance < 2 cm to the object and/or touching it with the nose. During the second trial (T_2_, 90 min after T_1_), one of the objects presented in trial T_1_ was replaced by a new object and the rats were left in the box for 5 min (Hazzaa et al., 2020). The time spent in the exploration of the familiar (F) and the new object (N) was recorded during the second trial.

Discrimination index (DI) was calculated using the formula; DI = (Time spent for Novel object exploration—Time spent for Familiar object exploration)/(Time spent for Novel object exploration + Time spent for Familiar object exploration). A positive value of DI, indicates the animal has taken more time investigating the novel object (Denninger et al., 2018).

#### 2.5.3 Biochemical estimation

On the eighth day, the animals were sacrificed by cervical dislocation. The brain was rapidly removed and refrigerated at 20°C. Using a handheld homogenizer (Remi Homogeniser, India), a 10% w/v of the brain tissue was homogenized with Tris-hydrochloric buffer (pH 7.4) The homogenized samples were centrifuged at 5000 rpm for 10 min. The sodium level in the brain was estimated using a commercial kit by Crest biosystems, which consists of sodium reagent and standard sodium (150 mEq/L). The calcium level was measured with the help of a commercial kit by Erba diagnostics consists of AMP (2-Amino-2-methy-1-propanol) reagent, OCPC (Ortho-cresolpthalein complexone) reagent and standard calcium (10 mg/dL) (Viswanatha Swamy et al., 2013).

#### 2.5.4 Histopathological studies

The brain was isolated after dissection and the cerebral cortex of the frontal lobe from each group were fixed using a 10% formalin. Then, the tissue specimens were embedded in paraffin. The frontal lobe blocks were cut into serial coronal sections (5 sections in each animal), 5 µm thickness. Haematoxylin and eosin were used for staining. The stained tissue was examined at ×10 and ×40 magnification using a light microscope (Viswanatha Swamy et al., 2013).

### 2.6 Statistical analysis

The results were expressed as mean ± SEM. Using a GraphPad Prism software, the results were analysed by a One-way ANOVA using Dunnett’s multiple comparison tests and *p*-values were calculated. *p* values ≤ 0.033 were considered as significant.

## 3 Results

**FIGURE 1:**
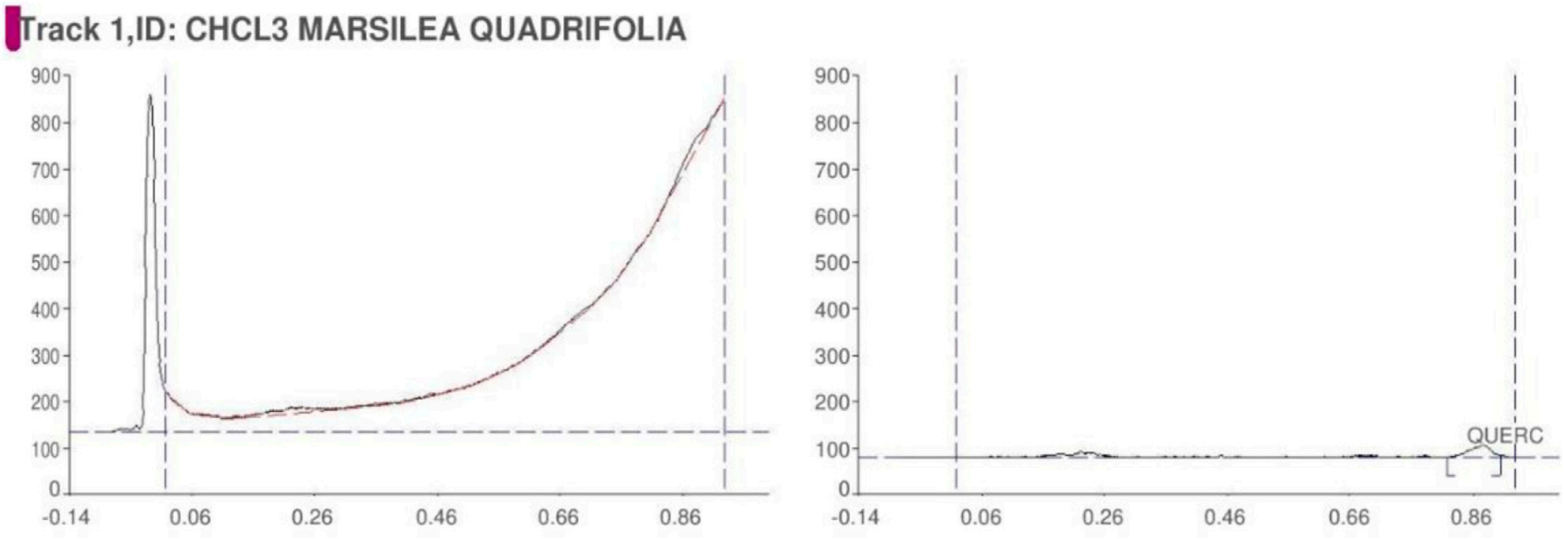
HPTLC of CEMQ.

**FIGURE 2:**
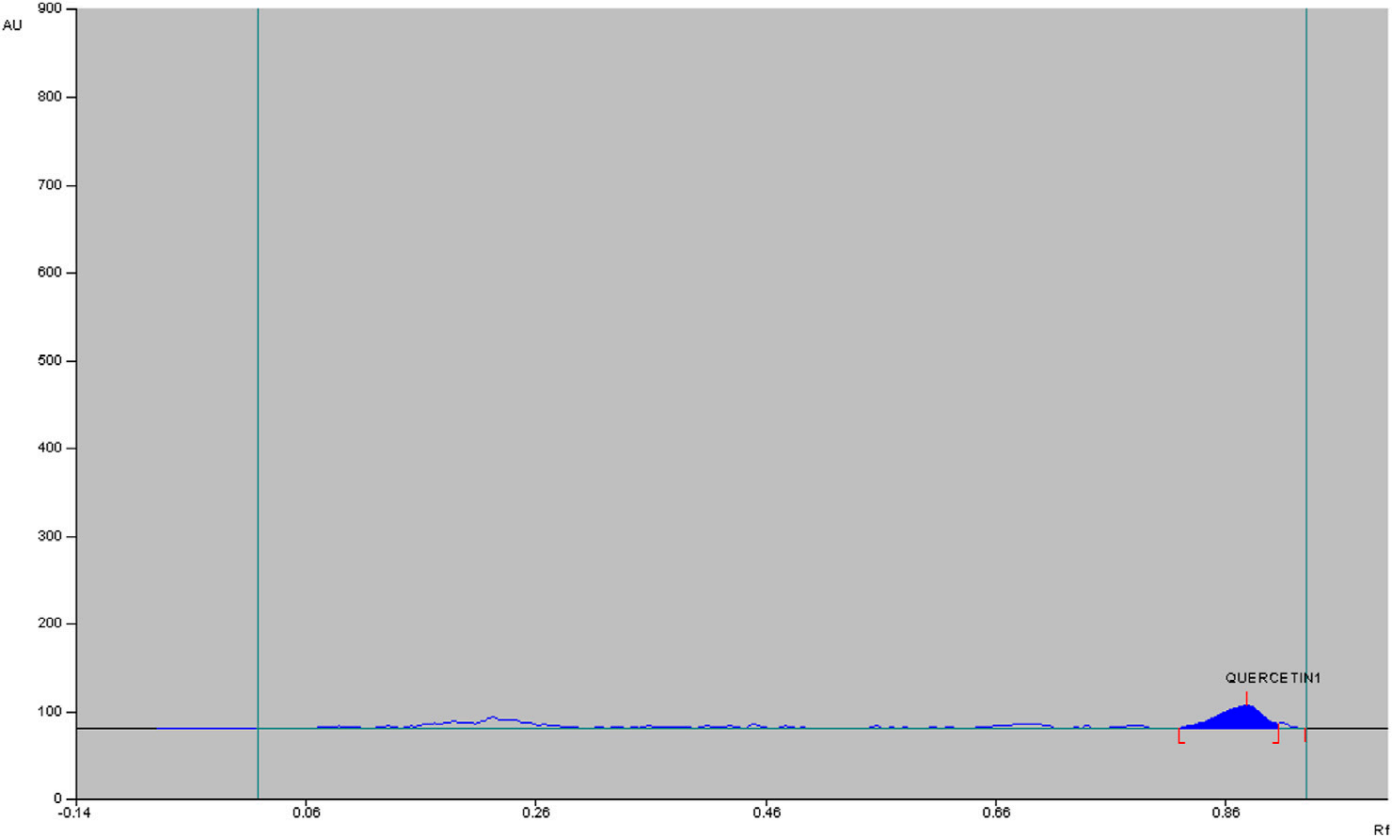
Chromatogram of CEMQ.

**FIGURE 3:**
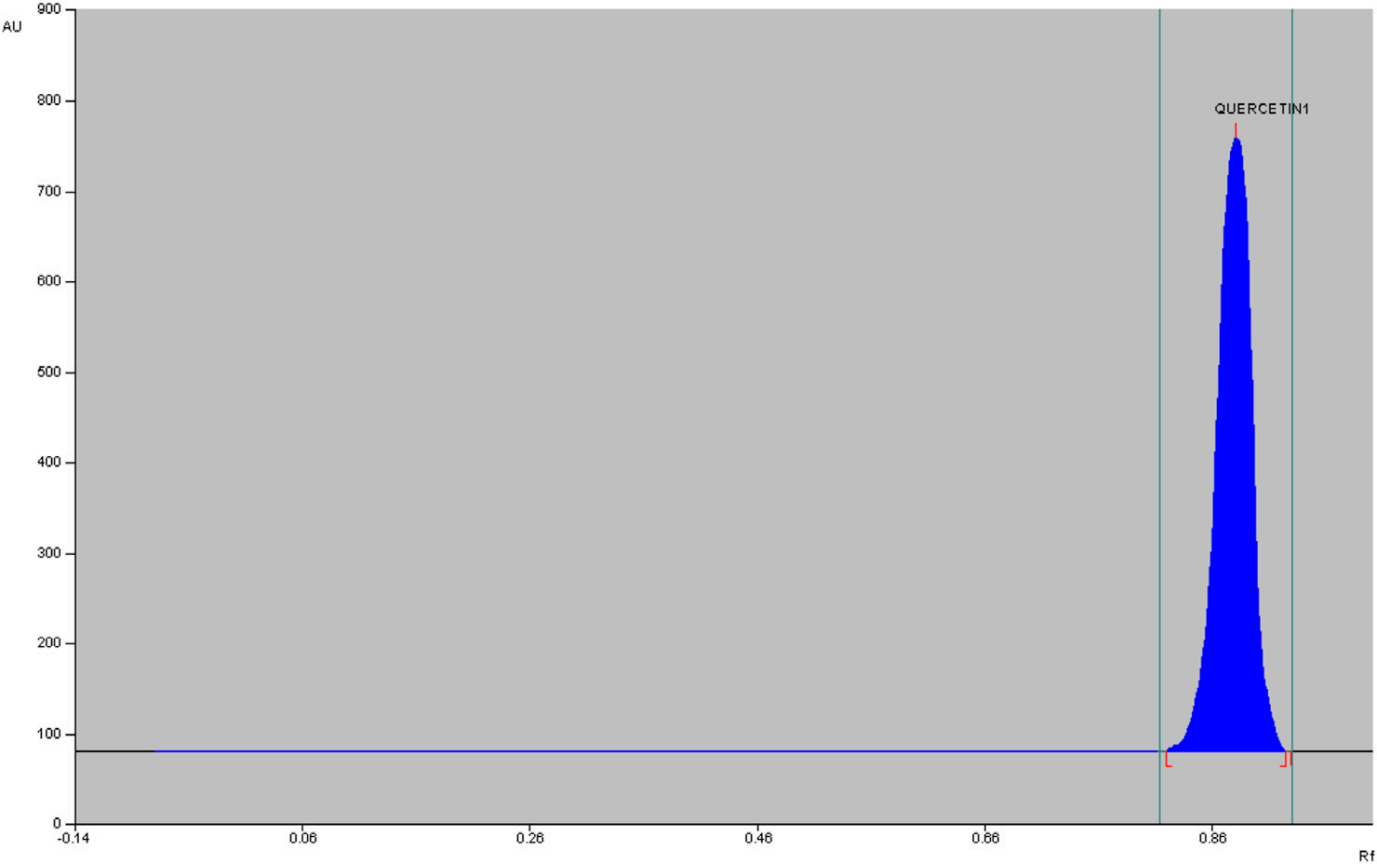
Chromatogram of standard quercetin.

The qualitative phytochemical analysis of CEMQ showed the presence of flavonoids and phenols. The percentage yield of CEMQ was higher (31.46% w/w) when compared to petroleum ether and ethanol extracts (12.02% and 21.36% w/w respectively). The HPTLC analysis of CEMQ revealed that it contains quercetin (Table 2; Figures 1–3).

**TABLE 2:** HPTLC data of CEMQ.

Peak	Starting R_f_	Starting Height	Max R_f_	Max Height	Max %	Ending R_f_	Ending Height	Area	Area %	Assigned substance
1	0.82	0.1	0.88	25.9	100	0.91	5.7	827.9	100	Quercetin

The manual calculation using the formula mentioned below indicated the presence of approximately 0.23% quercetin in CEMQ.

$$Manual\;calculation=\frac{Standard\;amount}{Standard\;area}\times Sample\;area$$

**FIGURE 4:**
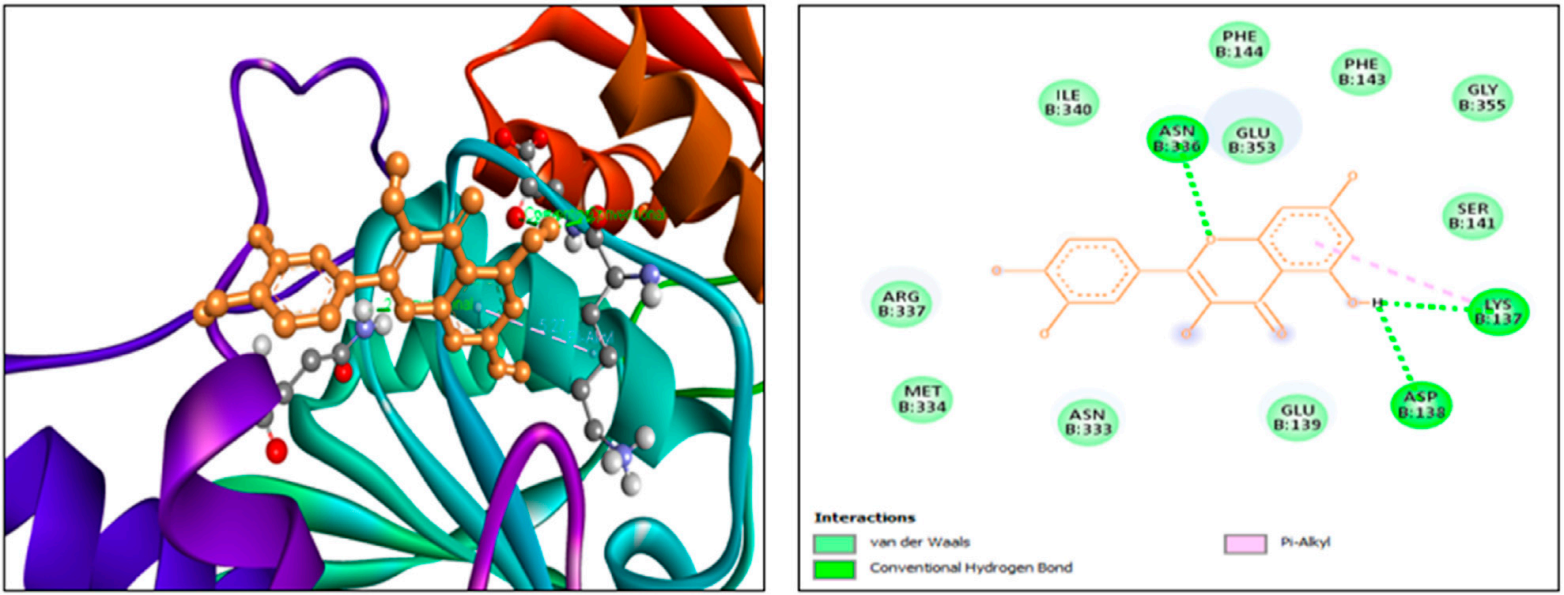
3D and 2D docking interactions of quercetin with 5EWJ.

**FIGURE 5:**
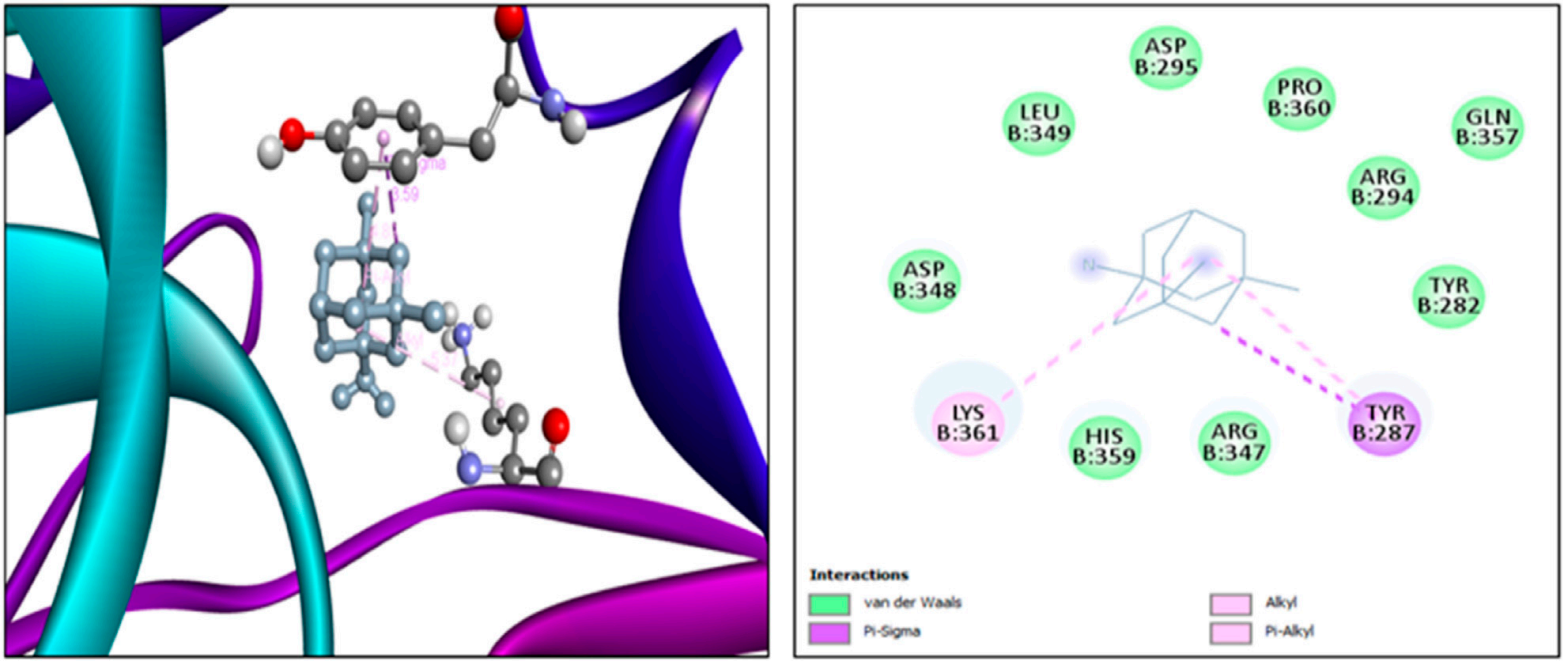
3D and 2D docking interactions of memantine with 5EWJ.

The findings from the *in silico* docking and the binding affinities were shown in Table 3 and described in Figures 4, 5.

**TABLE 3:** Molecular docking: binding scores and amino acid interactions.

S. No	Ligand structure	Ligand name	PUBCHEM ID	Binding affinity (kcal/mol)	Residue interaction
1	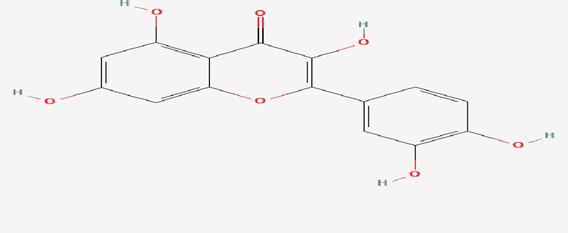	Quercetin	5280343	−7.9	ASN B:336, LYS B:137, ASP B:138
2	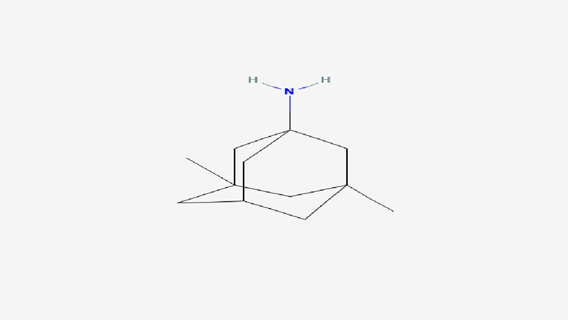	Memantine	4054	−6.2	LYS B:361, TYR B:287

The effect of CEMQ on the locomotor activity was shown in Table 4. Since their locomotion was greatly reduced, negative control animals were under an extreme oxidative stress, (Farombi and Onyema, 2006; Shivasharan et al., 2013), which may be contributed by the generation of free radicals following MSG administration. The animals that received the standard drug have significantly improved locomotory score when compared to negative control. The animals treated with 200 and 400 mg/kg of CEMQ also significantly increased the locomotory score when compared to the negative control. The locomotor activity of CEMQ treated groups was well comparable with the standard drug treated animals.

**TABLE 4:** Effect of CEMQ on locomotor activity using actophotometer in MSG-induced neurotoxicity.

S. No	Treatment	Locomotor activity score (counts/5 min)			
		Day 1	Day 3	Day 5	Day 7
1	Group I - Normal control: (1% w/v CMC, i.p.)	209.42 ± 0.31	210.31 ± 0.60	212.27 ± 0.24	213.62 ± 0.31
2	Group II - Negative control: MSG (2 g/kg, i.p.)	119.07 ± 0.19^###^	118.36 ± 0.28^###^	113.64 ± 0.44^###^	112.41 ± 0.80^###^
3	Group III - MSG (2 g/kg, i.p.) + Standard memantine (20 mg/kg, i.p.)	168.34 ± 0.40^***^	170.21 ± 0.17^***^	172.08 ± 0.68^***^	173.41 ± 0.20^***^
4	Group IV - MSG (2 g/kg, i.p.) + CEMQ (200 mg/kg, p.o.)	153.21 ± 0.46^***^	154.33 ± 0.81^***^	158.11 ± 0.43^***^	159.74 ± 0.47^***^
5	Group V - MSG (2 g/kg, i.p.) + CEMQ (400 mg/kg, p.o.)	158.51 ± 0.44^***^	158.08 ± 0.18^***^	161.71 ± 0.72^***^	164.30 ± 0.62^***^

All values were expressed as mean ± SEM (*n* = 6). The data were analyzed by a One-way ANOVA, using Dunnett’s multiple comparison tests; values are statistically significant at.

^###^ *p* < 0.001 between normal and negative control.

^***^ *p* < 0.001 between negative control and treated groups.

**FIGURE 6:**
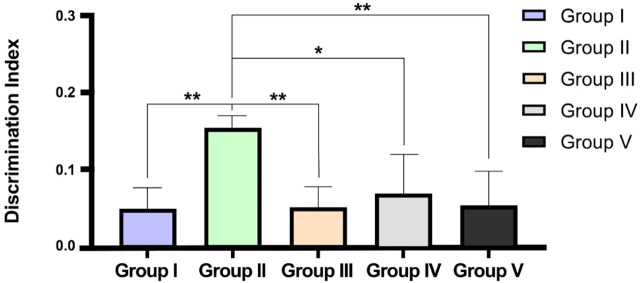
Effect on CEMQ on discrimination index using object recognition test. The data represents significance at *p < 0.003 and **p < 0.002.

The effect of CEMQ on non-spatial memory and learning using an object recognition and the manually calculated discrimination index was shown in Table 5 and Figure 6. The negative control animals administered with MSG showed a significant increase in the time spent to explore familiar and novel objects and showed a high positive value of calculated discrimination index, when compared to normal control animals indicating a reduction in the non-spatial learning and memory which may be attributed to neuro-inflammation. The standard drug treated animals showed improvement in memory and non-spatial learning ability since the time spent to explore both familiar and novel objects was significantly reduced. The CMEQ treated animals in both the doses (200 and 400 mg/kg) had a reduction in the object recognition time and had shown least positive in discrimination index calculation, when compared to the negative control group, indicating the potential neuroprotective activity.

**TABLE 5:** Effect of CEMQ on object recognition time in MSG-induced neurotoxicity.

S. No	Treatment	Time spent for object exploration (seconds)	
		Familiar object	Novel object
1	Group I—Normal control: (1% w/v CMC, i.p.)	30.15 ± 0.03	42.45 ± 0.10
2	Group II—Negative control: MSG (2 g/kg, i.p.)	65.04 ± 0.04^##^	80.32 ± 0.11^##^
3	Group III - MSG (2 g/kg, i.p.) + Standard memantine (20 mg/kg, i.p.)	38.15 ± 0.05^*^	45.21 ± 0.16^*^
4	Group IV - MSG (2 g/kg, i.p.) + CEMQ (200 mg/kg, p.o.)	43.24 ± 0.05^*^	50.76 ± 0.20^*^
5	Group V - MSG (2 g/kg, i.p.) + CEMQ (400 mg/kg, p.o.)	40.16 ± 0.01^*^	48.40 ± 0.05^*^

All values were expressed as mean ± SEM (*n* = 6). The data were analyzed by a One-way ANOVA, using Dunnett’s multiple comparison tests.

^##^ *p* < 0.002 between normal and negative control.

*p < 0.033 between negative control and treated groups.

The effect of CEMQ on the levels of biochemical parameters in MSG-induced neurotoxicity was shown in Table 6.

**TABLE 6:** Effect of CEMQ on levels of brain biochemical parameters in MSG-induced neurotoxicity.

S. No	Treatment	Sodium (mM)	Calcium (nM)
1	Group I - Normal control: (1% w/v CMC, i.p.)	8.64 ± 0.42	78.19 ± 1.01
2	Group II—Negative control: MSG (2 g/kg, i.p.)	14.05 ± 0.88^###^	139.62 ± 1.37^###^
3	Group III - MSG (2 g/kg, i.p.) + Standard memantine (20 mg/kg, i.p.)	9.13 ± 0.51^***^	88.03 ± 1.64^***^
4	Group IV - MSG (2 g/kg, i.p.) + CEMQ (200 mg/kg, p.o.)	10.91 ± 0.38^**^	107.51 ± 1.76^***^
5	Group V - MSG (2 g/kg, i.p.) + CEMQ (400 mg/kg, p.o.)	9.76 ± 0.62^***^	98.05 ± 1.51^***^

All values were expressed as mean ± SEM (n = 6). The data were analyzed by a One-way ANOVA, using Dunnett’s multiple comparison tests;

^###^ *p* < 0.001 between normal and negative control.

^***^ *p* < 0.001 and,

^**^ *p* < 0.002 between negative control and treated groups.

Electrolyte imbalance is a well-known fundamental mechanism causing cell death during brain damage. Dietary changes are thought to be a key factor in avoiding stroke. The risk of stroke may be impacted by dietary choices that change ionic or electrolyte levels. According to Moemeni et al. (2016), electrolyte may play a role in the brain’s deterioration. The biochemical parameters indicated a significant increase in sodium and calcium ions levels in negative control animals which received MSG (2 g/kg) for 7 days Standard drug treated group showed a reduction in sodium and calcium ions levels when compared to negative control animals due to the NMDA antagonistic effect of the drug. The CEMQ treated groups showed a similar biochemical response to that of standard drug treated which may be correlated with the antagonistic effect of CEMQ containing quercetin as well as quercetin derivatives on the NMDA receptor.

**FIGURE 7:**
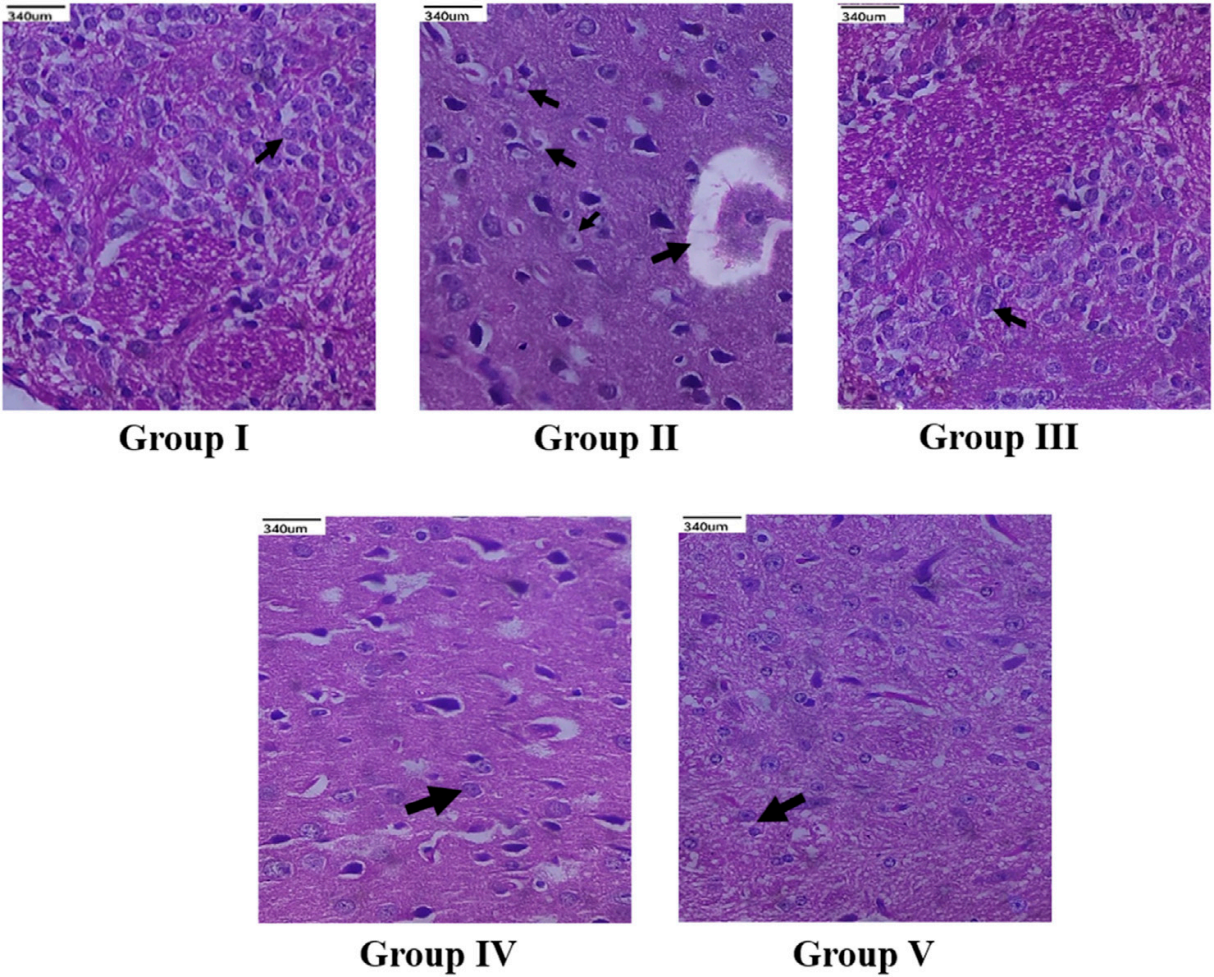
Histopathological changes (×40 magnification) of the cerebral cortex of frontal lobe region of brain. Group I (Normal control) showed normal neuronal cells with no morphological changes (indicated by arrow); Group II (Negative control) showed neuronal degeneration (indicated by arrow), loss of cellular integrity along with neuronal disorganization (indicated by arrow); Standard memantine-treated group III showed neuronal cells resembling normal neuronal cells (indicated by arrow), exhibiting neuroprotection by preventing further neuronal damage; CEMQ-treated group IV (200 mg/kg) showed definite number of neuronal cells (indicated by arrow), preventing against neuronal degeneration; CEMQ-treated group V (400 mg/kg) showed more definite number of neuronal cells (indicated by arrow) and less neuronal disorganization.

The histopathological study revealed that there were no morphological changes in the cerebral cortex of frontal lobe of the normal animals. No edema was seen and the cerebral cortex region was also normal. In Group II animals, pyknosis and karyorrhexis of nuclei were noted, along with cerebral edema, neuronal degeneration, and neuronal disorganization (Figure 7). The standard drug or memantine-treated Group III animals showed prevention of neuronal damage and indefinite appearance of pyknosis and karyorrhexis of nuclei. Groups IV and V animals also indicated neuronal protection against neuronal damage and less neuronal disorganization.

## 4 Discussion

*M. quadrifolia* is an edible aquatic medicinal plant used as a traditional health food in Asia (Zhang et al., 2016). It contains several vitamins and high amount of crude proteins (Abbasi et al., 2018). Further, it is anticipated that the CEMQ containing quercetin and its derivatives which are adequate to elicit a potential pharmacological action. Since, quercetin was reported to improve the health of experimental animals’ when used even in lower doses (Geng et al., 2019). Also, as indicated by Gopalakrishnan and Udayakumar (2017) and Zhang et al. (2016), the *M. quadrifolia* extracts may contain other quercetin derivatives and important phytoconstituents which may be responsible for significant adjuvant pharmacological actions.

The findings from *in silico* indicated that both quercetin and standard memantine exhibited excellent binding affinities with the NMDA glutamate receptor (5EWJ). The selected ligand (quercetin) exhibited a higher binding affinity against the NMDA glutamate receptor when compared to the standard ligand (memantine). In fact, quercetin showed NMDA glutamate receptor inhibitory potential to a similar degree as that of the standard drug, memantine which is an NMDA receptor antagonist.

The findings from the animal experiment indicated that the animals were more stressed due to the induced excitotoxicity as a result of the MSG administration, thus significantly reducing their locomotor score. It has also resulted in the impairment of non-spatial memory and learning of the animals and has contributed to an increase in calcium and sodium levels which may be attributed by the activation of NMDA receptors by MSG, causing ionic channels to open in an unregulated manner with excess entry of calcium and sodium ions (Sama and Norris, 2013; Abdel Moneim et al., 2018). Histopathological finding also indicated some evidence of neuronal damage like cerebral edema and neuronal necrosis which is a clear indication of MSG-induced neurotoxicity (Fardian et al., 2018).

The standard drug memantine (20 mg/kg. i.p.) conferred a neuroprotective property, since it is an NMDA antagonist, it improved the locomotor activity, memory and non-spatial learning while decreasing the levels of sodium and calcium ions (Lipton, 2006). Histopathological findings further confirmed amelioration of neurotoxicity as caused by MSG.

**FIGURE 8:**
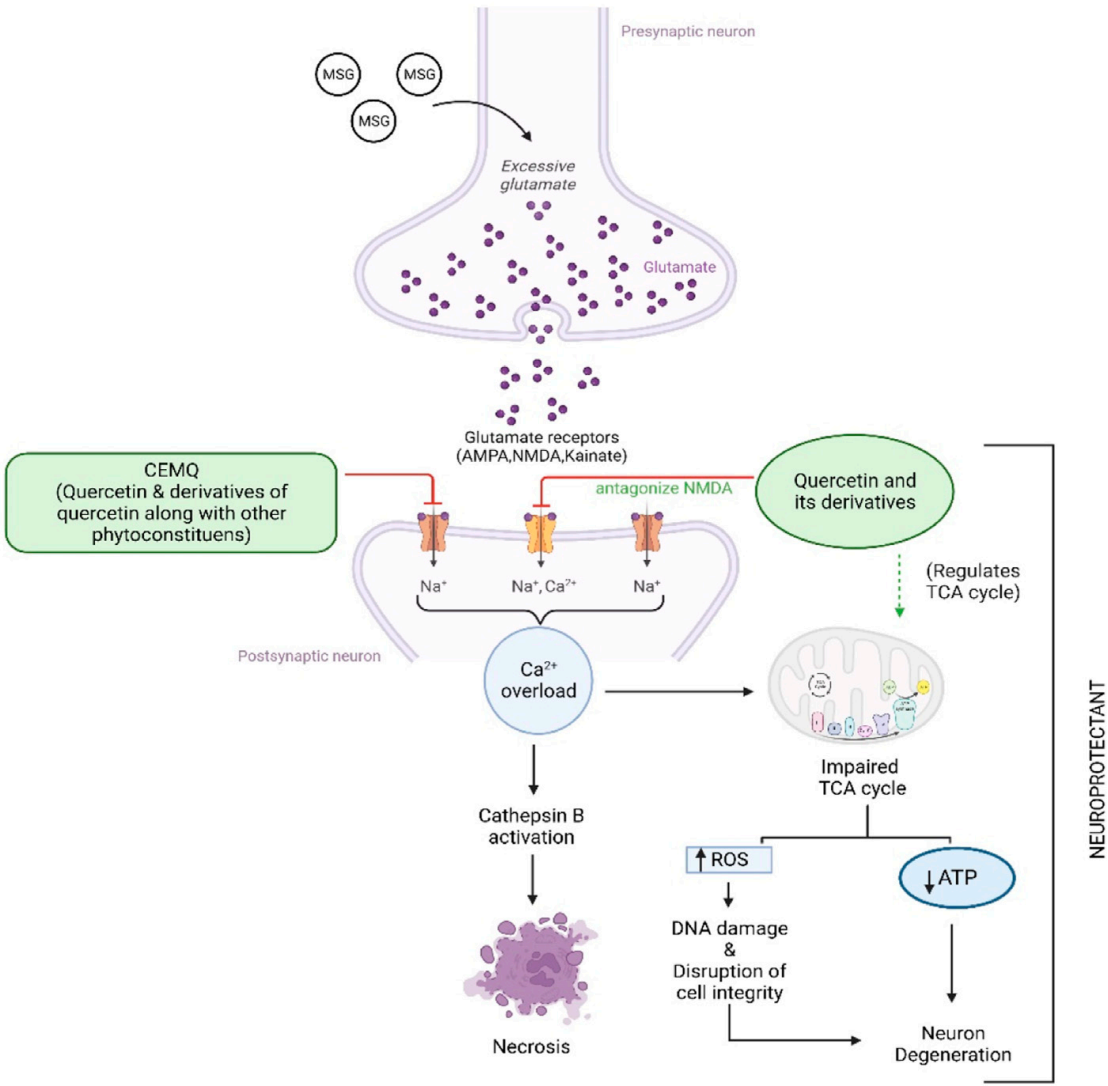
Proposed mechanism of CEMQ (containing quercetin and its derivatives) against MSG induced neurotoxicity.

The two doses of CEMQ (200 and 400 mg/kg) also produced significant effects as that of the standard drug, indicating that this extract can be used to prevent neurotoxicity in rats. The neurotrophic activity of the plant is attributed to quercetin, a potent anti-oxidant (Farombi and Onyema, 2006) that is also neuroprotective (Islam et al., 2021). Similarly, quercetin derivatives may be present in the extract, which may bind with the NMDA receptor and antagonize it, causing improvement against excitotoxicity caused by administered MSG (Figure 8). According to Zhang et al. (2016), *M. quadrifolia* is a potent source of polyphenols with potent antioxidant properties that may be utilised for alleviating oxidative stress. The field of antioxidant therapy has also made significant strides in recent studies, with a focus on neuroprotection in particular (Teleanu et al., 2019). Therefore, the antioxidant components of CEMQ, such as quercetin and its derivatives, may be responsible for the neuroprotective effects.

Modern food culture comprises the surplus use of food additives like Monosodium glutamate, Aspartame, that have been reported to cause excess generation of free radicals (Niaz et al., 2018; Tsatsakis et al., 2019; Das et al., 2022) resulting in the oxidative stress, which may lead to initiation and progression of many neurological conditions (Uttara et al., 2009). The plant extracts being rich in anti-oxidants could be a potential remedy to produce relief against many neurological conditions (Elufioye et al., 2017; Sharma et al., 2022).

To enhance memory and cognition, quercetin is utilised as a nutraceutical and dietary supplement (Dong et al., 2017). Quercetin can additionally protect against chronic illnesses including neurological diseases (Rubio-Ruiz et al., 2019). According to studies by Unsal et al. (2015), quercetin lowers the immunoreactivity of deteriorating neurons, promotes nerve cell recovery by reducing inflammatory responses, and aids in the recovery of motor function after spinal cord injury (Zhang et al., 2015). Additionally, quercetin has been recommended as a therapy option for such injury (Ocal et al., 2019) and has been shown to have a neuro-protective effect after acute spinal cord injury (Wang et al., 2018). The possible mechanism of quercetin against MSG-induced neurotoxicity has been depicted in Figure 7. The current study found quercetin in CEMQ, which might offer protection against the neurotoxicity caused by MSG.

### 4.1 Limitations of the study

The present study has focused on the short-term exposure rather than long-term exposure of MSG and the effect of MSG on other regions of brain was not explored, which has to be taken into consideration for future research works. In histopathology studies, much focus was not given on the layers of cerebral cortex, which must be taken into consideration for future studies. Further, confirmation of necrosis caused by MSG has to be taken into indication in upcoming studies with the help of suitable biomarkers. Apart from quercetin and its derivatives, other phytoconstituents which may be present in the extract might have an impact on the neuroprotective effect. Hence, further exploration of its pharmacognostic and phytochemical evaluations are necessary.

## 5 Conclusion

According to the findings of the current investigation, *M. quadrifolia*, a well-known plant with a long history of use, exhibits strong neuroprotective effect against the neurotoxicity caused by MSG. Excitotoxic neuronal damage and a greater production of reactive oxygen species are the main causes of MSG’s neurotoxicity. *M. quadrifolia* may be used in the prevention or potential therapy of neurological disorders due to its extensive antioxidant activity and high medicinal properties. CEMQ was shown to be potential neuroprotective activity against MSG-induced neurotoxicity in rats occurring in a dose-dependent manner. *M. quadrifolia* contain quercetin derivatives and some other phytoconstituents (antioxidants), acting similarly, to ameliorate excitotoxicity. Further research should be conducted on *M. quadrifolia* and CEMQ to reveal its phytochemicals that are responsible for neuroprotection.

## Data Availability

The original contributions presented in the study are included in the article/Supplementary material, further inquiries can be directed to the corresponding authors.

## Abbreviations

MSG, Monosodium glutamate; NMDA, N-methyl-D-aspartate; AMPA, α-amino-3-hydroxy-5-methyl-4-isoxazole-propionic acid; NDDs, Neurodegenerative diseases; CEMQ, Chloroform extract of Marsilea quadrifolia; HPTLC, High-Performance Thin Layer Chromatography; GABA, Gamma amino butyric acid.

## References

[B1] Abbasi S. A. Ponni G. Tauseef S. M. (2018). *Marsilea quadrifolia:* a new bioagent for treating wastewater. Water Air Soil Pollut. 229, 133. 10.1007/s11270-018-3743-z

[B2] Abdel Moneim W. M. Yassa H. A. Makboul R. A. Mohamed N. A. (2018). Monosodium glutamate affects cognitive functions in male albino rats. Egypt. J. Forensic Sci. 8, 9. 10.1186/s41935-018-0038-x

[B3] Afifi M. Abbas A. (2011). Monosodium glutamate versus diet induced obesity in pregnant rats and their offspring. Acta Physiol. hung. 98, 177–188. 10.1556/APhysiol.98.2011.2.9 21616776

[B4] Bhadra S. Mukherjee P. K. Bandyopadhyay A. (2012). Cholinesterase inhibition activity of Marsilea quadrifolia Linn. an edible leafy vegetable from West Bengal, India. Nat. Prod. Res. 26, 1519–1522. 10.1080/14786419.2011.565006 21978132

[B5] Bhargava A. Shrivastava P. Tilwari A. (2021). HPTLC analysis of Fumaria parviflora (Lam.) methanolic extract of whole plant. Futur. J. Pharm. Sci. 7, 1. 10.1186/s43094-020-00150-x

[B6] Chauhdary Z. Saleem U. Ahmad B. Shah S. Shah M. A. (2019). Neuroprotective evaluation of Tribulus terrestris L. in aluminum chloride induced Alzheimer’s disease. Pak. J. Pharm. Sci. 32, 805–816. Available at: http://www.ncbi.nlm.nih.gov/pubmed/31103976 . 31103976

[B7] Creeley C. Wozniak D. F. Labruyere J. Taylor G. T. Olney J. W. (2006). Low doses of memantine disrupt memory in adult rats. J. Neurosci. 26, 3923–3932. 10.1523/JNEUROSCI.4883-05.2006 16611808 PMC6673894

[B8] Das D. Banerjee A. Bhattacharjee A. Mukherjee S. Maji B. K. (2022). Dietary food additive monosodium glutamate with or without high-lipid diet induces spleen anomaly: A mechanistic approach on rat model. Open Life Sci. 17, 22–31. 10.1515/biol-2022-0004 35128066 PMC8802345

[B9] Denninger J. K. Smith B. M. Kirby E. D. (2018). Novel object recognition and object location behavioral testing in mice on a budget. J. Vis. Exp. 10.3791/58593 PMC6800058 30531711

[B10] Dong F. Wang S. Wang Y. Yang X. Jiang J. Wu D. (2017). Quercetin ameliorates learning and memory via the Nrf2-ARE signaling pathway in D-galactose-induced neurotoxicity in mice. Biochem. Biophys. Res. Commun. 491, 636–641. 10.1016/j.bbrc.2017.07.151 28757412

[B11] Dong X. Wang Y. Qin Z. (2009). Molecular mechanisms of excitotoxicity and their relevance to pathogenesis of neurodegenerative diseases. Acta Pharmacol. Sin. 30, 379–387. 10.1038/aps.2009.24 19343058 PMC4002277

[B12] Elufioye T. O. Berida T. I. Habtemariam S. (2017). Plants-derived neuroprotective agents: cutting the cycle of cell death through multiple mechanisms. Evidence-Based Complement. Altern. Med. 2017, 3574012–3574027. 10.1155/2017/3574012 PMC5585568 28904554

[B13] Fardian N. Maulina M. La Tabari M. F. Mardiati (2018). “The effect of monosodium glutamate (msg) administration to pyramidal cells necrosis on cerebral cortex of wistar male rats (*Rattus norvegicus*),” in Proceedings of MICoMS 2017, 187–192. 10.1108/978-1-78756-793-1-00070

[B14] Farombi E. O. Onyema O. O. (2006). Monosodium glutamate-induced oxidative damage and genotoxicity in the rat: modulatory role of vitamin C, vitamin E and quercetin. Hum. Exp. Toxicol. 25, 251–259. 10.1191/0960327106ht621oa 16758767

[B15] Geng L. Liu Z. Wang S. Sun S. Ma S. Liu X. (2019). Low-dose quercetin positively regulates mouse healthspan. Protein Cell 10, 770–775. 10.1007/s13238-019-0646-8 31325157 PMC6776572

[B16] Gomathi D. Ravikumar G. Kalaiselvi M. Vidya B. Uma C. (2012). HPTLC fingerprinting analysis of Evolvulus alsinoides (L.) L. *J. Acute Med.* 2, 77–82. 10.1016/j.jacme.2012.08.004

[B17] Gopalakrishnan G. Udayakumar R. (2017). Phytochemical content of leaf and stem of Marsilea quadrifolia (L.). J. Plant Sci. Phytopathol. 1, 026–037. 10.29328/journal.jpsp.1001003

[B18] Hajihasani M. M. Soheili V. Zirak M. R. Sahebkar A. Shakeri A. (2020). Natural products as safeguards against monosodium glutamate-induced toxicity. Iran. J. Basic Med. Sci. 23, 416–430. 10.22038/IJBMS.2020.43060.10123 32489556 PMC7239414

[B19] Hazzaa S. M. Abdelaziz S. A. M. Abd Eldaim M. A. Abdel-Daim M. M. Elgarawany G. E. (2020). Neuroprotective potential of allium sativum against monosodium glutamate-induced excitotoxicity: impact on short-term memory, gliosis, and oxidative stress. Nutrients 12, 1028. 10.3390/nu12041028 32290031 PMC7230314

[B20] Heath P. R. Shaw P. J. (2002). Update on the glutamatergic neurotransmitter system and the role of excitotoxicity in amyotrophic lateral sclerosis. Muscle Nerve 26, 438–458. 10.1002/mus.10186 12362409

[B22] Islam M. S. Quispe C. Hossain R. Islam M. T. Al-Harrasi A. Al-Rawahi A. (2021). Neuropharmacological effects of quercetin: A literature-based review. Front. Pharmacol. 12, 665031. 10.3389/fphar.2021.665031 34220504 PMC8248808

[B23] Jenila Bejads X. Antro Jennie X. Rajesh M. Srinivasa Kumar K. P. (2014). Evaluation of anti-inflammatory activity of methanol extract of the plant *Marsilea quadrifolia* on albino rats using carrageenan and histamine induced paw edema. J. Glob. Trends Pharm. Sci. 5, 1640–1644. https://www.jgtps.com/admin/uploads/CZsqX5.pdf .

[B24] Khalil R. M. Khedr N. F. (2016). Curcumin protects against monosodium glutamate neurotoxicity and decreasing NMDA2B and mGluR5 expression in rat Hippocampus. Neurosignals 24, 81–87. 10.1159/000442614 27529496

[B25] Lipton S. A. (2006). Paradigm shift in neuroprotection by NMDA receptor blockade: memantine and beyond. Nat. Rev. Drug Discov. 5, 160–170. 10.1038/nrd1958 16424917

[B27] Merve Bayram H. Fatih Akgoz H. Kizildemir O. Ozturkcan A. (2023). Monosodium glutamate: review on preclinical and clinical reports. Biointerface Res. Appl. Chem. 13, 149. 10.33263/BRIAC132.149

[B28] Moemeni H. Qujeq D. Ahangar A. A. Hajian K. Parsian H. (2016). Evaluating the serum levels of calcium, chloride, potassium and sodium in the stroke patients. Int. J. Med. Laboratory 3 (2), 104–110. http://ijml.ssu.ac.ir/article-1-110-en.html .

[B29] Niaz K. Zaplatic E. Spoor J. (2018). Extensive use of monosodium glutamate: A threat to public health? EXCLI J. 17, 273–278. 10.17179/excli2018-1092 29743864 PMC5938543

[B30] Ocal O. Borcek A. O. Pasaoglu O. Gundogdu A. C. Kaplanoglu G. T. Baykaner M. K. (2019). Can quercetin be an option for treatment of spinal cord injury? An experimental study. Turk. Neurosurg. 29 (2), 247–253. 10.5137/1019-5149.JTN.23799-18.1 30649798

[B31] OECD (2022). Test No. 425: Acute oral toxicity: Up-and-Down procedure. OECD. 10.1787/9789264071049-en

[B32] Parambi D. G. T. Saleem U. Shah M. A. Anwar F. Ahmad B. Manzar A. (2020). Exploring the therapeutic potentials of highly selective oxygenated chalcone based MAO-B inhibitors in a haloperidol-induced murine model of Parkinson’s disease. Neurochem. Res. 45, 2786–2799. 10.1007/s11064-020-03130-y 32939670

[B34] Rousseaux C. G. (2008). A review of glutamate receptors I: current understanding of their biology. J. Toxicol. Pathol. 21, 25–51. 10.1293/tox.21.25

[B35] Rubio-Ruiz M. E. Guarner-Lans V. Cano-Martínez A. Díaz-Díaz E. Manzano-Pech L. Gamas-Magaña A. (2019). Resveratrol and quercetin administration improves antioxidant defenses and reduces fatty liver in metabolic syndrome rats. Molecules 24 (7), E1297. 10.3390/molecules24071297 PMC6479544 30987086

[B36] Sahu S. Dutta G. Mandal N. Goswami A. R. Ghosh T. (2012). Anticonvulsant effect of Marsilea quadrifolia Linn. On pentylenetetrazole induced seizure: A behavioral and EEG study in rats. J. Ethnopharmacol. 141, 537–541. 10.1016/j.jep.2012.02.039 22414476

[B37] Sama D. M. Norris C. M. (2013). Calcium dysregulation and neuroinflammation: discrete and integrated mechanisms for age-related synaptic dysfunction. Ageing Res. Rev. 12, 982–995. 10.1016/j.arr.2013.05.008 23751484 PMC3834216

[B38] Shah H. Naseer A. Gupta N. Singh R. (2022). A study on HPTLC quantification for quality control of myricetin a nutraceutical from different plant parts of myrica esculenta Linn. Biomed. Pharmacol. J. 15, 219–228. 10.13005/bpj/2357

[B39] Sharma K. Verma R. Kumar D. Nepovimova E. Kuča K. Kumar A. (2022). Ethnomedicinal plants used for the treatment of neurodegenerative diseases in Himachal Pradesh, India in Western Himalaya. J. Ethnopharmacol. 293, 115318. 10.1016/j.jep.2022.115318 35469830

[B40] Shivasharan B. D. Nagakannan P. Thippeswamy B. S. Veerapur V. P. (2013). Protective effect of Calendula officinalis L. Flowers against monosodium glutamate induced oxidative stress and excitotoxic brain damage in rats. Indian J. Clin. biochem. 28, 292–298. 10.1007/s12291-012-0256-1 24426226 PMC3689330

[B41] Singh S. A. Dhanasekaran D. Ganamurali N. Preethi L. Sabarathinam S. (2021). Junk food-induced obesity-a growing threat to youngsters during the pandemic. Obes. Med. 26, 100364. 10.1016/j.obmed.2021.100364 34580647 PMC8459649

[B42] Singh S. Mishra S. B. Mukerjee A. (2021). “HPTLC fingerprinting analysis of phytoconstituents from indigenous medicinal plants,” in Evidence based validation of traditional medicines (Singapore: Springer Singapore), 337–358. 10.1007/978-981-15-8127-4_17

[B43] Stroebel D. Buhl D. L. Knafels J. D. Chanda P. K. Green M. Sciabola S. (2016). A novel binding mode reveals two distinct classes of NMDA receptor GluN2B-selective antagonists. Mol. Pharmacol. 89, 541–551. 10.1124/mol.115.103036 26912815 PMC4859819

[B45] Subramanian A. Tamilanban T. Alsayari A. Ramachawolran G. Wong L. S. Sekar M. (2022). Trilateral association of autophagy, mTOR and Alzheimer’s disease: potential pathway in the development for Alzheimer’s disease therapy. Front. Pharmacol. 13, 1094351. 10.3389/fphar.2022.1094351 36618946 PMC9817151

[B46] Subramanian M. Balakrishnan S. (2019). Anti-venom activities of methanol extract of Marsilea quadrifolia Linn (Marsileaceae) against Russell’s Viper venom. Int. J. Res. Pharm. Sci. 10, 1540–1546. 10.26452/ijrps.v10i2.873

[B47] Teleanu R. I. Chircov C. Grumezescu A. M. Volceanov A. Teleanu D. M. (2019). Antioxidant therapies for neuroprotection-A review. J. Clin. Med. 8 (10), 1659. 10.3390/jcm8101659 31614572 PMC6832623

[B48] Tsatsakis A. M. Docea A. O. Calina D. Buga A. M. Zlatian O. Gutnikov S. (2019). Hormetic Neurobehavioral effects of low dose toxic chemical mixtures in real-life risk simulation (RLRS) in rats. Food Chem. Toxicol. 125, 141–149. 10.1016/j.fct.2018.12.043 30594548

[B49] Unsal C. Kanter M. Aktas C. Erboga M. (2015). Role of quercetin in cadmium-induced oxidative stress, neuronal damage, and apoptosis in rats. Toxicol. Ind. Health 31 (12), 1106–1115. 10.1177/0748233713486960 23645211

[B50] Uttara B. Singh A. Zamboni P. Mahajan R. (2009). Oxidative stress and neurodegenerative diseases: A review of upstream and downstream antioxidant therapeutic options. Curr. Neuropharmacol. 7, 65–74. 10.2174/157015909787602823 19721819 PMC2724665

[B33] Venkatachalam G. Balasundaram J. (2017). Evaluation of diuretic activity of ethanolic extract of leaves of *Marsilea quadrifolia* Linn. Eur. J. Pharm. Med. Res. 4, 458–461. https://storage.googleapis.com/journal-uploads/ejpmr/article_issue/1509363340.pdf .

[B51] Viswanatha Swamy A. H. M. Patel N. L. Gadad P. C. Koti B. C. Patel U. M. Thippeswamy A. H. M. (2013). Neuroprotective activity of pongamia pinnata in monosodium glutamate-induced neurotoxicity in rats. Available at: www.ijpsonline.com . PMC3928729 24591740

[B52] Wang Y. Li W. Wang M. Lin C. Li G. Zhou X. (2018). Quercetin reduces neural tissue damage and promotes astrocyte activation after spinal cord injury in rats. J. Cell. Biochem. 119 (2), 2298–2306. 10.1002/jcb.26392 28865131

[B53] Zhang Y. Tian H.-Y. Tan Y.-F. Wong Y.-L. Wu H. Y. Jia J.-F. (2016). Isolation and identification of polyphenols from Marsilea quadrifolia with antioxidant properties *in vitro* and *in vivo* . Nat. Prod. Res. 30, 1404–1410. 10.1080/14786419.2015.1062377 26222269

[B54] Zhang Y. Yi B. Ma J. Zhang L. Zhang H. Yang Y. (2015). Quercetin promotes neuronal and behavioral recovery by suppressing inflammatory response and apoptosis in a rat model of intracerebral hemorrhage. Neurochem. Res. 40, 195–203. 10.1007/s11064-014-1457-1 25543848

